# Pneumonia

**Published:** 1855-11

**Authors:** Wm. Van Pelt

**Affiliations:** Williamsville, Erie County, N. Y.


### ART. III.—*Pneumonia.* By Wm. Van Pelt, M. D., Williamsville, Erie County, N. Y.

The recent discussions, by the Buffalo Medical Association, on the subject of pneumonia, have afforded much interest. The different views entertained in regard to the importance and propriety of venesection, the value of observing the buffy coat of the drawn blood, and the abstraction of blood after solidification has supervened, are worthy of notice, as affording indications of the wide difference in practice which may be found in one small city.

If anything can be said to be established in the practice of the medical art, it is that anti-phlogistic remedies are the only proper means by which acute phlegmasiæ can be successfully subdued, and suppuration or effusion prevented, and that pneumonia particularly demands venesection to effect a perfect cure.

Such are the teachings in our medical schools, and such are the instructions of all systematic writers on physic. In connection, it is worthy of remark, that about the only places where this general rule can be fully carried out, is in hilly or mountainous countries, in which intermittent and remittent fevers are seldom seen. It is undoubtedly worse than folly to abandon general principles; but the adoption of no treatment is quite a different thing from applying an imperfect one. Soda, Varrentrap, and Dietl, in the treatment of pneumonia, claim even a greater amount of success, than that of the usual course, by very simple means. Notwithstanding their almost no treatment may have been equal to the successful management of the disease as it is found in their respective locations, I imagine it would be difficult to prove that a judicious medication would not have hastened the recovery, in many of those instances that were left to the power of nature, for such was essentially the fact.

It is no new idea that certain local causes exert an influence over the diseases developed within their sphere. It is probably true that New York, Buffalo, and very many other places, may have peculiarities, terrene and atmospherical, that will justify frequent departures from the ordinary course, similar to that followed by Prof. Metcalfe. But that method adopted as general, will undoubtedly be attended with a proportionable number of failures, because all the cases presented will not be of a character to be relieved by it as they might be were the usual method employed.

Due allowance must always be made for the peculiar views presented by authors, and claimed as being most proper, because the individual experience in a particular place, establishes them in opinion thus; therefore the method must be world-wide in application. Medical literature abounds in instances of the sort.

The admission that venesection is proper in severe cases, is a powerful argument for its employment in a proportionate degree in the milder. The propriety of its use, or other means, must be a matter for the medical attendant to decide as circumstances of condition appear to demand.

Neither is it a question, of how many cures can be effected in any given number, by following any particular method; but rather what will best restore this individual case to a normal condition. Such is the general problem presented.

It is affirmed that the power of self-limitation in pneumonia, in contradistinction to the general teachings, will cure nearly all cases, and instead of the malady being of a nature to threaten life, it is a very trivial affair. It is also claimed to furnish an easy explanation why the success that attends opposite methods of practice, have been about equal, which, by the by, has not been established. It is not easy to understand how any one could be justified in saying that other measures than those adopted, would have done better or worse, particularly when an equal success attends. The tendency is to reduce practice to a uniform standard; or rather to establish principles for general application. Exceptions there must be, and it is to be expected that exceptions will be more difficult to understand, and furnish more particular points for study than the general rule. No writer pretends to explian the *modus operandi* by which miasma affects the human organization in all places within its sphere of influence. The miasmata originating intermittent and remittent fever, yet remains undefined in its constitution. We only know that certain topographical conformations of country maintain peculiar pyrexiæ, and modify its phlegmasiæ. No truth in medicine is better established. If we needed an instance, illustrative, I would adduce the mixed practice, that obtains within a circle of fifty miles around Buffalo. As you approach the mountainous country to the south, and south-east, you will find the uniformity in practice to increase in regard to the treatment of thoracic affections. Pneumonitis among the hills, in Buffalo, and the once Tonawanda swamp, is not the same disease, requiring the same management. In the former, repeated venesection, and all other antiphlogistic means, are required; in the latter, it becomes often but little more than congestion, and the bloody sputa, and sense of tightness, are the occasion of calling the attention. The recumbent posture, rest, diet, counter-irritation, diaphoretics and laxatives, will remove it. In a large share of the cases the intermittent type is so plainly visible that no one hesitates to prescribe quinine; and it will generally remove the physical signs indicating the presence of pneumonia, with an astonishing rapidity. The obstacle to the adoption of a uniform method of treatment in this locality, is the occurrence of about ten per cent. of the cases in which the pure unmodified inflammatory character is manifest; they demand an energetic antiphlogistic treatment, which local experience fully justifies. These, tonics invariably exasperate. In proof that this method of treatment is required, may be adduced the perfection of the cures, the immediate cessation of the pain, and the expectoration of bloody sputa, and the instantaneous improvement that follow a proper venesection, such as is manifest after the employment of no other remedy.

Between these extremes, we have an infinite degree of complexure that make it no small task to adapt the treatment to the two-fold nature of the malady. The proneness of certain diseases to "anastomose" or wear each other's livery, is remarked by many writers. It is shown that when the paroxysm of intermittents, and its kindred diseases, suffer a "transfer of functions," the emunctories refusing to act so as to bring about a crisis, the internal organs are affected with congestions and inflammations. It is hardly proper to call them inflammations, because they are so different in a therapeutical aspect.

Although attended with heat, redness and tumefaction, nevertheless they are not so liable to suppurate, as idiopathic inflammation. They are a distinct species of inflammation, isopathic with intermittent fever.

Occasionally writers, living in places in which intermittents are common, introduce peculiar notions, which can be satisfactorily accounted for by a clear, geographic, and climatic knowledge pertaining to that count y. This apparently discordant practice can be made to harmonize.

At times it is quite impossible to distingnish inflammations of this modified character, so closely do they simulate the pure variety; particularly, phrenitis, pneumonia, and dysentery. Not unfrequently this similarity has been the cause of bitter disputes between physicians, because they were not aware of the nature of the complication. It is to an error in this respect that the great mortality that attended the entrance of the French army into Algiera, is to be attributed; the surgeons supposed it was gastro-cephalitis they had to treat, and adopted severe antiphlogistic means, when they should have followed an opposite plan.

It would seem, in this view of the question, that we need some other pathognomonic symptoms in addition, to guide unerringly in relation to these characteristics. Very often the tending to periodicity and exalted neuralgic character of the pain which belongs to the intermittent form, afford sufficient indications. We think the physical signs accompanying pneumonia and pulmonic affections of the intermittent type, are not so marked in degree, that the solidification in pneumonia is not so firm, and the stages into which the disease, on account of utility, is divided, are more protracted in duration.

The buff is quite a sure guide in the real or acute pneumonia; but fails just in proportion as the case is influenced by that unknown, mysterious miasma. The less firm solidification, all circumstances being equal, would naturally lead to the opinion that venesection would be more beneficial in the intermittent variety; but it is not so; undoubtedly the power which produced the transfer of functions, also affects the absorbents so as to prevent the same degree of absorption of the lymph, that unquestionably takes place in pure pneumonia after a judicious venesection.

We have, in accordance to certain conditions in disease, the terms *sthenic* and *asthenic*, which indicate the character of any case to which they may be applied; but they throw no light on the mysterious difference or parallelism which exists. Neither is it certain but that they generally show the presence, or otherwise, of these same troublesome miasmatic influences.

After all that can be said, the universal test by which the necessary course of treatment to be adopted in epidemics, is to borrow a phrase from another science, by "trial and error." Consequently an increase of mortality will follow a change in the "epidemic constitution," for the reason that the adopted practice is not simultaneously changed in its coördinate relations to answer the new condition. Did we possess more accurate knowledge in regard to the laws governing miasma, and their power to modify disease, we should not have the mortification of readingnotices which proclaim the disgrace of medicine, such as appears in the Bulletin Genéral de Therapeutique, as follows, to wit: "Within the last six months there have appeared a number of cases of masked aguish neuralgia, which so simulate other diseases as not infrequently to be mistaken." From time to time, similar statements appear from other sources equally respectable.

It appears, therefore, in relation to this subject, that it needs a revision, which may consume years in its accomplishment; but if successful it is not too much to say, that in many respects the art of medicine will merit a claim to perfection beyond most of the sister sciences.

Being perfectly aware that there is nothing new presented in the foregoing remarks, I am constrained to make them because some gentlemen, of no mean capacities, entertain views in relation to pneumonia, so utterly at variance with my own, as your Journal reports show.

WILLIAMSVILLE, Sept. 30, 1855.

